# Indonesia national registry of children with type 2 diabetes mellitus

**DOI:** 10.1186/1687-9856-2013-S1-P8

**Published:** 2013-10-03

**Authors:** Annang Giri Moelyo, Indra W Himawan, Vivekenanda Pateda, Ryadi Fadil

**Affiliations:** 1 Sebelas Maret University, Surakarta, Central Java, Indonesia; 2 Lambung Mangkurat University, Banjarmasin, Indonesia; 3 Sam Ratulangi University, North Sulawesi, Indonesia; 4 Padjadjaran University, West Java, Indonesia

## Background

There was increasing number of children with diabetes mellitus in Indonesia (based on Indonesia National Registry). Amount of Indonesia children with Type 1 Diabetes Mellitus (T1DM) reach more than eight hundred, but it is still unknown for Type 2 Diabetes Mellitus (T2DM).

## Aim

To know the number of Indonesia children with T2DM and their characteristics.

## Methods

A national survey of T2DM children in Indonesia was conducted in April 2009-March 2012. All members of Indonesia Pediatric Endocrinology Chapter were involved for this study. Epidemiology data were collected by a registry form. The form consists of age, gender, age at diagnosis, A1c level at diagnosis, risk factors (obese, family history) and therapy.

## Results

There are 38 children with T2DM (16 boys, 22 girls). Twenty two children are diagnosed at >10 years old. Cases come spreadly from 11 provinces, the most cases are from Jakarta (16), Central Java (7) and West Java (6). Most frequent cases are diagnosed in May, June and December. Almost all cases are diagnosed by Pediatric Endocrinologist (10 cases). Twenty children are obese. Only 10 children had weight loss and 1 child had ketoasidosis. Mean BMI at diagnosis is 24 (+2.2) kg/m2. Mean of A1c at diagnosis is 11.5% (+ 1.9). Therapy at diagnosis are oral hypoglycemic only (4), insulin only (11), combination of insulin and oral hypoglycemic (1). Diabetes family histories are found in 16 subjects, (1 T1DM and 15 T2DM); 4 subjects have no family history.

## Conclusion

Incidence of Indonesia children with T2DM less than T1DM. Almost all data are from Jakarta as capital city. It should be larger number of cases because of increasing number of obese children. Awareness and better registry system must be increased. Obese and diabetes family history are the risk factors for Indonesia children with T2DM.

